# MVO: A Magneto-Visual Odometry System for Indoor Positioning

**DOI:** 10.3390/s26144555

**Published:** 2026-07-17

**Authors:** Tongxing Peng, Chao Ming, Zhengpeng Yang, Huaiyan Wang, Jiyan Yu, Xiaoming Wang

**Affiliations:** College of Mechanical Engineering, Nanjing University of Science and Technology, Nanjing 210094, China; 221101024477@njust.edu.cn (T.P.); yzp_njust@njust.edu.cn (Z.Y.); yujiyan@139.com (J.Y.); 202xm@163.com (X.W.)

**Keywords:** magnetometer array, magneto-visual odometry, indoor positioning, magnetic field-based localization, iSAM2, factor graph optimization

## Abstract

High-precision and resilient indoor positioning is a fundamental requirement for the autonomous operation of mobile robots in GNSS-denied environments. While visual sensors are commonly used for odometry, their operational reliability can be compromised in challenging scenarios such as drastic illumination fluctuations and sparse-textured environments. To address these sensor limitations, this study presents MVO, a magneto-visual odometry framework that explores indoor magnetic field anomalies as complementary constraints for visual odometry. By integrating a 30-magnetometer planar array model with a stereo camera, the proposed system establishes a multi-modal perception framework for indoor spaces. In the frontend, magnetic field gradient information is utilized to provide relative-pose constraints, which assist in the matching of image feature points and help maintain tracking continuity under visual degradation. In the backend, a factor graph optimization (FGO) framework incorporates magnetic relative-pose factors and visual reprojection factors into a unified optimization objective, which is then solved using the incremental smoothing and mapping 2 (iSAM2) algorithm. Frontend-level simulations are conducted to analyze the effects of magnetometer spatial configuration, sensor number, and calibration-error sensitivity on magnetic relative-pose estimation and covariance. Trajectory-level evaluations are further performed on the EuRoC dataset augmented with high-fidelity synthesized magnetic field data, including localization accuracy and computational load. Under this synthesized magnetic field setting, MVO shows improved localization accuracy and moderate computational load compared with the selected MSCKF-Stereo and VINS-Fusion reference baselines. These results provide a simulation-based feasibility validation of integrating magnetic field constraints with visual information for indoor odometry, while validation with real magnetometer array measurements remains future work.

## 1. Introduction

Pose information is among the most critical data required for mobile robots to achieve full autonomy. Outdoors, the integration of the global navigation satellite system (GNSS) with the inertial measurement unit (IMU) offers an efficient and high-precision solution. However, a large number of mobile robots operate in indoor environments, where satellite signals remain unreliable due to obstruction by building structures.

Indoor positioning has therefore been studied through both infrastructure-based localization and onboard odometry. Wi-Fi round-trip time (RTT) and fine-timing measurement (FTM) methods can exploit commercial access points and mobile devices for meter-level indoor positioning [1]. Visible light positioning (VLP) based on visible light communication (VLC) and location fingerprinting can further achieve centimeter-level accuracy, and recent VLP studies have improved robustness under ambient light variations and reduced fingerprinting complexity [2,3]. However, these methods typically depend on deployed access points or light-emitting diode (LED) infrastructure, known transmitter locations, fingerprint databases, line-of-sight conditions, or environment-specific calibration. For mobile robots that require continuous pose estimation during motion, onboard odometry remains essential and complementary to infrastructure-based positioning.

For indoor positioning of mobile robots, visual observations are commonly employed to develop odometry systems [4,5,6,7]. Nevertheless, a standalone visual approach often suffers from significant drift or even complete failure when facing challenges in degraded scenarios—such as extreme variations in illumination intensity and insufficient visual features. Currently, the most widely adopted strategy to mitigate this problem is to fuse visual measurements with inertial data, thereby constructing visual-inertial odometry (VIO). A range of advanced VIO algorithms have been proposed [8,9,10,11]; these algorithms improve the robustness and accuracy of odometry systems by integrating inertial measurements. Yet, they cannot fully resolve the issue of system drift or failure when visual outage occurs. This is primarily because low-cost IMUs are universally used in indoor mobile robots: during visual outages, the biases of the IMU accumulate rapidly during the integration process, leading to substantial pose errors.

Most indoor workspaces for robots are man-made environments, and the abundance of ferromagnetic materials in such settings disrupts the spatial distribution of the magnetic field. The magnetic field generated by these ferromagnetic materials superimposes on the Earth’s magnetic field, resulting in a non-uniform magnetic field with distinct gradients. This unique magnetic field can serve as a spatial position reference for the robot [12,13]. Unlike IMUs, which depend on sensing the robot’s self-motion for integration, magnetic-field-based localization provides an external reference for the carrier. In principle, this enables magnetic-field-based localization to reduce the rapid buildup of cumulative errors. Additionally, magnetic fields are unaffected by illumination conditions, making them a potential complementary cue when visual perception is degraded. Thus, fusing visual information with magnetic field data may further enhance the accuracy and robustness of odometry systems in indoor environments. In this work, we investigate this idea in a synthesized magnetic field setting as an initial feasibility study.

This paper proposes a magneto-visual odometry system, with the following specific contributions:

(1) We derive the model-based magnetic odometry frontend, and on this foundation, propose a feature matching mechanism that leverages the magnetic frontend to assist the visual frontend—effectively enhancing the reliability of feature association in visually degraded scenarios.
(2) We propose a novel magneto-visual backend optimization framework built upon factor graph optimization (FGO) and incremental smoothing and mapping 2 (iSAM2), which enables unified fusion of visual and magnetic measurement information while maintaining computational efficiency.
(3) We evaluate the proposed approach at both the frontend and trajectory levels: frontend simulations analyze magnetometer array configuration, sensor number, calibration error sensitivity, and relative-pose covariance, while trajectory-level experiments on public visual datasets with synthesized magnetic field data assess localization accuracy and computational load.

The remainder of the work is organized as follows. Section 2 is a review of related work. Section 3 offers a system overview of the proposed method, and Section 4 details the derivation of the odometry system’s frontend encompassing both the magnetic frontend and visual frontend. Section 5 describes the system’s backend optimization framework. Section 6 presents the magnetic frontend analysis, trajectory-level localization evaluation, and computational load evaluation. Section 7 summarizes our contributions and discusses future work.

## 2. Related Work

Visual information is a major source of navigation data in GNSS-denied indoor environments. Early vision-based state estimation can be traced back to Moravec et al. [14], and later systems such as Oriented FAST and Rotated BRIEF Simultaneous Localization and Mapping (ORB-SLAM) [6] demonstrated accurate localization and mapping by extracting and tracking rotation-invariant visual features. However, pure visual odometry (VO) remains sensitive to illumination changes, sparse texture, motion blur, and fast motion. Visual-inertial odometry (VIO) addresses part of this limitation by fusing visual and inertial measurements. Representative filter-based methods include the multi-state constraint Kalman filter (MSCKF) [15], which constrains the camera trajectory using multi-state feature observations. Subsequent work improved consistency and observability using approaches such as the estimated Jacobian-EKF [16], unscented Kalman filter variants [17], and tightly coupled EKF/UKF formulations [18]. Nevertheless, when visual information is strongly degraded, low-cost IMU integration can still introduce rapid drift because inertial biases accumulate during visual outages.

Infrastructure-based techniques form another important category of indoor positioning methods. Wi-Fi RTT/FTM positioning estimates distance from time-of-flight measurements between commercial Wi-Fi devices and access points, with accuracy affected by ranging-bias calibration, access point geometry, and signal propagation conditions [1]. Visible light positioning (VLP) uses LED transmitters and optical receivers, and fingerprinting-based VLP can achieve high accuracy when the light-source layout and fingerprint database are well constructed [2]. Recent VLP studies further improve robustness to ambient light interference and reduce fingerprinting complexity through robust signal processing and sparse reconstruction [3]. These methods are valuable for indoor localization, but their dependence on deployed infrastructure or pre-built environmental information makes them complementary to self-contained odometry systems for mobile robots.

Indoor magnetic fields provide another source of environmental information. Ferromagnetic materials in buildings make the local magnetic field spatially non-uniform, creating location-dependent magnetic anomalies that can be used for localization. Skog et al. [12] proposed a model-based magnetic odometry method that fits a polynomial local magnetic field model from magnetometer array measurements and estimates motion by minimizing magnetic field residuals. Skog et al. later introduced an optical-flow-inspired magnetic odometry formulation [13]. Huang et al. [19] further fused magnetic and inertial measurements in the extended square root Kalman filter (ESKF) framework, forming a magnetometer-assisted inertial navigation system (MAINS). Compared with visual sensing, magnetometers are insensitive to illumination and can provide useful cues in texture-poor regions where magnetic gradients are present. These properties motivate the fusion of magnetic and visual information for indoor odometry.

## 3. System Overview

**Figure 1:**
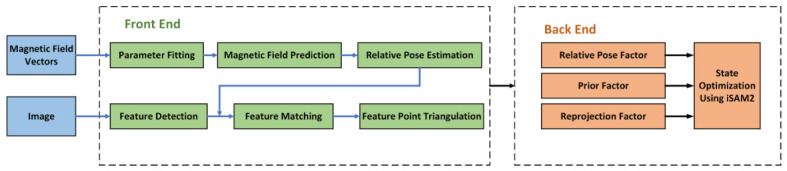
System overview of MVO. The frontend processes image data and magnetic field measurements to obtain the relative pose and 3D feature points, while the backend utilizes information from the frontend to construct a factor graph and optimize the carrier state.

The system proposed in this paper is mainly divided into two components, the frontend and the backend, which are shown in Figure 1: the frontend receives measurement data from the magnetometer array and the camera, where the magnetometer data are used for parameter fitting of the local magnetic field model and magnetic field prediction to ultimately estimate the relative pose, while the image data first undergo feature detection to extract feature points, then uses the relative pose information estimated from the magnetic field to assist in feature matching, and finally triangulates the matched feature points before sending both the estimated relative pose and the triangulated feature points to the backend for optimization; in the backend, relative pose factors and reprojection factors are constructed based on the relative pose and feature points output by the frontend, and these factors, together with the prior factors derived from the initial state, form a factor graph, which undergoes incremental updates via the iSAM2 [20] algorithm.

## 4. Front-End

In this section, we implement the acquisition of information for the front-end of the odometry system, which mainly consists of two parts. First, the acquisition of relative poses based on the magnetic array is realized. Second, we perform image processing that includes feature detection and magnetic-field-aided feature tracking.

### 4.1. Magnetic Array Based Relative Poses Acquisition

Following the model-based magnetic odometry formulation in [12,13], the local magnetic field is modeled under Maxwell’s equations. In the absence of free currents within the local workspace Ω, the magnetic field exhibits curl-free and divergence-free properties. Specifically, let $r={\left[x,y,z\right]}^{⊤}$ denote a 3D position in the local body frame, $M\left(r;\mu \right)\in R^{3}$ denote the magnetic field vector at *r*, and μ denote the coefficient vector of the polynomial magnetic field model. The following conditions must be satisfied for all r∈Ω:

(1a) $$\begin{array}{cc} \nabla_{r}\times & M(r;\mu )=0 \end{array}$$

(1b) $$\begin{array}{cc} \nabla_{r}\cdot & M(r;\mu )=0 \end{array}$$

Define ϕ(r;μ) as a scalar potential function. As indicated by (1a), M(r;μ) is the gradient of ϕ(r;μ), that is,

(2) $$M\left(r;\mu \right)=\nabla_{r}\phi \left(r;\mu \right).$$

Further, the scalar potential function is modeled as a polynomial of order (l+1), thereby obtaining the magnetic field model function in the form of a polynomial of order *l* [12]:

(3) $$M\left(r;\mu \right)=\nabla_{r}\phi \left(r;\mu \right)=\Gamma \left(r\right)\mu .$$

Here, Γ(r) denotes the polynomial basis matrix obtained from the gradient of the scalar potential function, μ is the corresponding coefficient vector, and *l* is the order of the magnetic field polynomial model. For example, the form of Γ when l=1 is as follows:

(4) $$\Gamma \left(r\right)=\left[\begin{array}{ccccccccc} 1 & 0 & 0 & y & z & 0 & 2x & 0 & 0\\ 0 & 1 & 0 & x & 0 & z & 0 & 2y & 0\\ 0 & 0 & 1 & 0 & x & y & 0 & 0 & 2z \end{array}\right].$$

In addition, the magnetic field model is simultaneously constrained by (1b), and combining Equations (1b) and (3) yields the following:

(5) $$\nabla_{r}\cdot \Gamma \left(r\right)\mu =\sum_{i=x,y,z}\frac{d{\left[\Gamma \left(r\right)\mu \right]}_{i}}{dr_{i}}=0\;\forall r\in \Omega .$$

This constraint can be written as the linear equation system:

(6) Dμ=0

where *D* is the coefficient matrix induced by the divergence-free constraint.

Under the constraint of (6), the current model now fully satisfies the curl-free and divergence-free conditions. Following [12], this model can be reparameterized as a lower-order model by introducing the matrix $D^{⊥}≜\mathrm{null}\left\{D\right\}$, whose columns span the null space of *D*. Let $\mu =D^{⊥}\theta$ and $\Phi \left(r\right)=\Gamma \left(r\right)D^{⊥}$, where θ is the reduced model parameter vector and Φ(r) is the constrained magnetic field basis. A reparameterized lower-order model for the magnetic field is given by:

(7) M(r;θ)=Φ(r)θ.

Once the polynomial-form magnetic field model is obtained, the coefficient vector of the magnetic field model in the body frame of the *k*th state can be estimated using measurements from the magnetic array as follows [12]:

(8) $${\hat{\theta }}_{k}={\left(\sum_{i=1}^{L}\Phi^{⊤}\left(d^{\left(i\right)}\right)\Phi \left(d^{\left(i\right)}\right)\right)}^{-1}\sum_{i=1}^{L}\Phi^{⊤}\left(d^{\left(i\right)}\right)y_{k}^{\left(i\right)}.$$

Here, *L* denotes the number of magnetometers in the array, $d^{\left(i\right)}$ is the fixed position of the *i*th magnetometer in the array/body frame, and $y_{k}^{\left(i\right)}\in R^{3}$ denotes the three-axis magnetic measurement of the *i*th magnetometer at the *k*th state.

**Figure 2:**
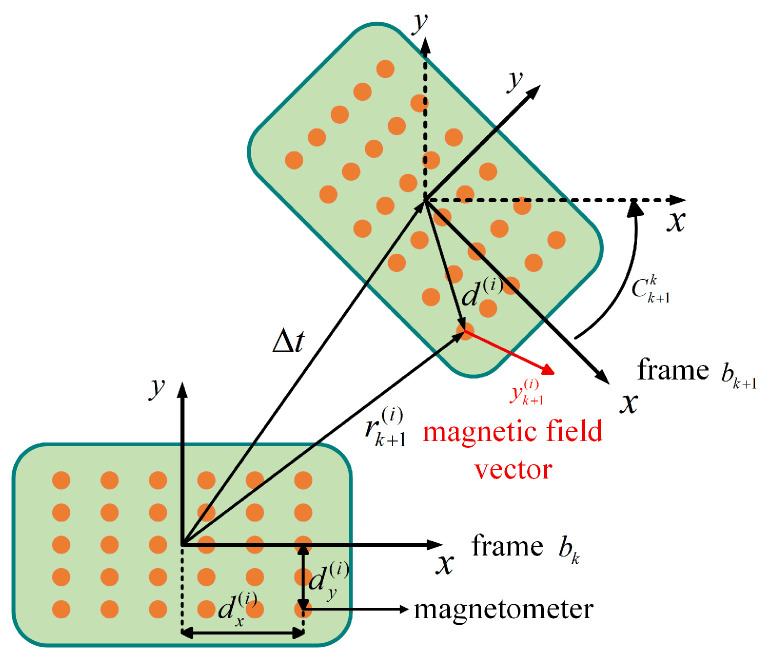
Schematic diagram of coordinate system definition for two consecutive measurements.

By obtaining the magnetic field model of the *k*th state, the relationship of the relative pose of the *k*th state with respect to the (k+1)th state and the estimate of the magnetic field measurements corresponding to the (k+1)th state can be modeled. Coordinate definitions and interrelationships between variables are as illustrated in Figure 2.

To estimate the (k+1)th state’s magnetic field measurements using the *k*th state’s magnetic field model, the sensor position in frame $b_{k+1}$ must be transformed to frame $b_{k}$. Let $r_{k+1}^{\left(i\right)}$ denote the position of the *i*th magnetometer in the array expressed in frame $b_{k}$ at the (k+1)th state. It can be expressed using the fixed sensor position $d^{\left(i\right)}$, the relative translation vector $t_{k+1}^{k}$, and the rotation matrix $C_{k+1}^{k}$ between the two states as follows:

(9) $$r_{k+1}^{\left(i\right)}=C_{k+1}^{k}d^{\left(i\right)}+t_{k+1}^{k}.$$

Here, $t_{k+1}^{k}$ denotes the position of frame $b_{k+1}$ expressed in frame $b_{k}$, and $C_{k+1}^{k}$ denotes the rotation matrix from frame $b_{k+1}$ to frame $b_{k}$. The magnetic field measurement estimate of the (k+1)th state ${\hat{y}}_{k+1}^{\left(i\right)}$ can be expressed as:

(10) $${\hat{y}}_{k+1}^{\left(i\right)}={C_{k+1}^{k}}^{⊤}\Phi \left(r_{k+1}^{\left(i\right)}\right){\hat{\theta }}_{k}.$$

Let the number of magnetometers on the array be *L*. The total vector of magnetic field measurement for the array $y_{k+1}$ is given by:

(11) $$y_{k+1}={\left[\begin{array}{c} y_{k+1}^{\left(1\right)}\ldots y_{k+1}^{\left(L\right)} \end{array}\right]}^{⊤}.$$

As an estimate of $y_{k+1}$, ${\hat{y}}_{k+1}$ also takes the same form.

After the estimate vector ${\hat{y}}_{k+1}$ is obtained, we can establish a residual equation using this estimate and the actual measurement vector $y_{k+1}$, consistent with model-based magnetic relative-pose estimation [12,13]. Let $\xi_{k+1}^{k}=\left(t_{k+1}^{k},C_{k+1}^{k}\right)$ denote the relative pose variable to be optimized. The residual objective is as follows:

(12) $$V\left(\xi_{k+1}^{k}\right)={∥y_{k+1}-h\left(\xi_{k+1}^{k}\right)∥}^{2}.$$

Here, $h(\xi_{k+1}^{k})$ denotes the magnetic field estimate

(13) $$\begin{array}{cc} h(\xi_{k+1}^{k}) & ={\bar{C}}_{k+1}^{k}\left[\begin{array}{c} \Phi (r_{k+1}^{\left(1\right)})\\ \vdots \\ \Phi (r_{k+1}^{\left(L\right)}) \end{array}\right]{\hat{\theta }}_{k},\\ {\bar{C}}_{k+1}^{k} & =I_{L}⊗{C_{k+1}^{k}}^{⊤}. \end{array}$$

Finally, the pose estimation problem can be formulated as a nonlinear least squares optimization problem:

(14) $${\hat{\xi }}_{k+1}^{k}=\arg\min_{\xi_{k+1}^{k}}V\left(\xi_{k+1}^{k}\right).$$

The relative pose can be obtained by solving the optimization problem.

### 4.2. Image Processing

As a visual-based odometry system, visual feature points are also required in the front-end. In a visual odometry (VO) system, the acquisition of feature points generally involves two sequential steps: the first is to detect feature points within the field of view, and the second is to track these feature points across image frames.

First, the features from accelerated segment test (FAST) feature detector is employed for feature detection [21]. It rapidly identifies corner-like features in images by assessing pixel intensity differences, while optimizing efficiency via a pre-test and non-maximum suppression.

Second, for the implementation of pose constraints, it is necessary to track the detected features across consecutive frames as well as between the left and right cameras.

#### 4.2.1. Magnetic-Field-Aided Feature Tracking

To conserve computational resources, feature tracking between consecutive frames is performed only on the sequential images from the left camera, also referred to as camera 0. Since the relative pose between consecutive frames has been estimated from the magnetic array measurements before image processing, this information can be used to assist visual feature tracking. If feature depths are known, the feature positions in the previous frame can be predicted in the current frame using the relative pose. Because the feature depths are not yet available at this stage, we assume a unit depth for all feature points. The 3D coordinates $X_{k-1}$ of the feature points in the previous frame can then be expressed as:

(15) $$X_{k-1}=K^{-1}\left[\begin{array}{c} u_{k-1}\\ v_{k-1}\\ 1 \end{array}\right].$$

Here, *K* is the intrinsic parameter matrix of the camera, and $u_{k-1}$ and $v_{k-1}$ denote the pixel coordinates of the point projected onto the camera image plane. Subsequently, the 3D coordinates of the feature points in the current frame can be calculated using the relative pose obtained earlier:

(16) $$X_{k}=C_{k-1}^{k}\left(X_{k-1}-t_{k}^{k-1}\right)$$

where $t_{k}^{k-1}$ denotes the relative translation from the previous frame to the current frame, expressed in the previous frame. The pixel coordinate of the feature in the *k*th state is then obtained by projection as:

(17) $$\left[\begin{array}{c} u_{k}\\ v_{k}\\ 1 \end{array}\right]∼KX_{k},$$

where ∼ denotes equality up to a homogeneous scale.

Once the prediction of feature points in the current frame is completed, their predicted pixel coordinates can serve as initial values for feature tracking. Specifically, we adopt the Lucas–Kanade–Tomasi (LKT) [22] optical flow method for feature point tracking, where high-quality initial values contribute to enhanced efficiency and accuracy of the tracking process.

#### 4.2.2. Stereo Match

Similarly, feature tracking between the left and right cameras within the same frame is also implemented using the LKT method. Given that the extrinsic parameters between the binocular cameras are known, outliers can be rejected based on the epipolar constraint after optical flow tracking is completed. The essential matrix between the left and right cameras is

(18) $$E=\left\lfloor t\times \right\rfloor R$$

Here, $\left\lfloor t\times \right\rfloor$ denotes the skew-symmetric matrix of the translation vector between the left and right cameras, and *R* denotes the external parameter rotation matrix between the left and right cameras. The epipolar line equation in the right camera can be expressed using the essential matrix:

(19) l=Ep

where $p={\left[u,v,1\right]}^{⊤}$. Furthermore, the epipolar constraint error of the feature points tracked in the right camera can be evaluated using the following formula:

(20) $$\mathrm{error}=\frac{{∥p^{\prime }l∥}_{1}}{\sqrt{l_{x}^{2}+l_{y}^{2}}}.$$

By evaluating this error, outliers that do not satisfy the epipolar constraint can be removed.

Through feature detection and subsequent feature tracking, the visual sensor supplies the odometry system with feature points that maintain stable tracking across multiple frames. In contrast to the front-end information derived from the magnetic field, these visually tracked feature points impose constraints on the carrier’s motion over a larger scale range.

## 5. Back-End

**Figure 3:**
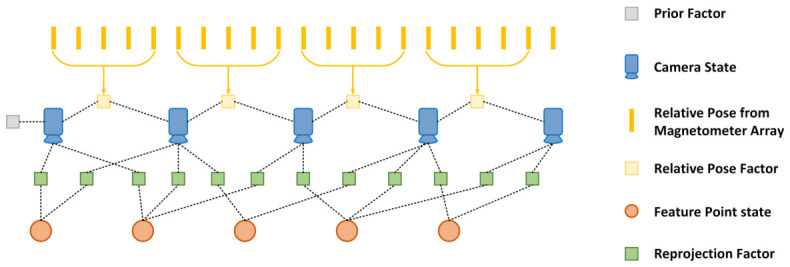
MVO backend factor graph structure. Dotted lines indicate factor connections. The three factor types added to the graph are the relative pose factor, reprojection factor, and prior factor.

This section focuses on the back-end processing of the magnetic field and visual front-end information obtained in the previous section. First, a factor graph optimization framework is established, and factors are constructed using the front-end information. Second, the generation of factors, as shown in Figure 3, is discussed.

### 5.1. Factor Graph Optimization Framework

The core task of an odometry system is state estimation, which refers to the process of inferring states from observations. In accordance with Bayes’ theorem, the posterior probability of the state X given the observation Z can be expressed as:

(21) $$P\left(X|Z\right)=\frac{P(Z|X)P(X)}{P(Z)}.$$

Subsequently, the state estimation problem can be modeled as a Maximum A Posteriori (MAP) estimation problem. Formally, the estimate is given by

(22) $$\begin{array}{cc} X^{MAP} & =\arg\max_{X}P\left(X|Z\right)\\ \; & =\arg\max_{X}P\left(Z|X\right)P\left(X\right) \end{array}$$

Here, P(Z∣X) denotes the likelihood probability and P(X) denotes the prior probability. Maximizing this posterior is equivalent to minimizing the negative log-posterior, which leads to the least-squares form used in factor graph optimization. Furthermore, in an odometry system, common states include the carrier’s state and the landmark’s state. Due to the Markov property of the carrier’s state, the state prior P(X) can be further factorized as

(23) $$P\left(X\right)=P\left(X_{0}\right)\prod_{k=0}^{T-1}P\left(X_{k+1}|X_{k}\right).$$

Meanwhile, due to the independence of different observations, the likelihood probability P(Z∣X) can also be factorized as

(24) $$P\left(Z|X\right)=\prod_{k=1}^{T}P\left(Z_{k}|X_{k}\right).$$

After factorizing the posterior probability, a factor graph can be constructed on the basis of the relationship between states and observations.

As illustrated in Figure 3, the proposed odometry system comprises two types of states and three types of factors. Specifically, the states encompass the camera state and the feature point state: the camera state—also referred to as the carrier state—includes the carrier’s position and orientation within the reference frame; the feature point state, which corresponds to the landmark state defined earlier, denotes the 3D position of each feature point in the reference frame. Both of these states serve as variables to be optimized in the proposed odometry system and are constrained by the factors. The three types of factors are the prior factor, the relative pose factor, and the reprojection factor, respectively. Among these factors, the prior factor serves to provide initial constraints on the system states, thereby ensuring the stability and uniqueness of the optimization process. Specifically, the relative pose factor and reprojection factor correspond to constraints derived from magnetic field measurements and visual measurements, respectively. In the subsequent sections, we will elaborate on the construction methods for the relative pose factor and reprojection factor.

### 5.2. Relative Pose Factor

Typically, the output frequency of the relative poses derived from the measurements of the magnetometer array is higher than the image frame rate. Consequently, to construct the relative pose factor between two consecutive image frames, it is first necessary to accumulate all relative pose measurements obtained during the interval between these two frames. As illustrated in Figure 3, we obtain the relative pose between two consecutive image frames by accumulating the relative pose from the magnetometer array:

(25) $$T_{k+n}^{k}=\prod_{i=1}^{n}T_{k+i}^{k+i-1}.$$

Here, $T_{k+n}^{k}$ denotes the accumulated relative pose from frame k+n to frame *k* and can be written as

(26) $$T_{k+n}^{k}=\left[\begin{array}{cc} C_{k+n}^{k} & t_{k+n}^{k}\\ 0 & 1 \end{array}\right].$$

Following this, the relative pose factor can be constructed using the relative pose obtained. As shown in (24), the relative pose factor corresponds to the factorized likelihood probability; therefore, the relative pose factor between the *k*th and (k+n)th frames can be modeled as follows:

(27) $$P\left(z_{k}^{k+n}|x_{k},x_{k+n}\right)\propto \exp\left(-\frac{1}{2}{∥e_{k}^{k+n}∥}_{\Sigma_{k}^{k+n}}^{2}\right).$$

The covariance $\Sigma_{k}^{k+n}$ defines the weight of the magnetic relative pose factor. It is not treated as an arbitrary constant; instead, it is obtained from the uncertainty of the magnetic frontend. Let $r_{B}$ denote the stacked magnetic-field residual of the magnetometer array, $J_{\xi }=\partial r_{B}/\partial \xi$ denote the Jacobian of the residual with respect to the six-DoF relative-pose perturbation ξ, and $\Sigma_{\epsilon }$ denote the covariance of the magnetic residual. The residual covariance contains both the uncertainty of the fitted local magnetic-field model and the magnetometer measurement noise, which can be written as

(28) $$\Sigma_{\epsilon }=\bar{R}\Sigma_{M}{\bar{R}}^{T}+\sigma_{m}^{2}I,$$

where $\Sigma_{M}$ is the covariance of the magnetic field model prediction estimated from the fitting residuals, $\bar{R}$ is the block-diagonal rotation matrix that transforms the model uncertainty into the measurement frame, and $\sigma_{m}$ is the standard deviation of the magnetometer measurement noise. For each magnetic relative-pose estimate, the corresponding pose covariance is approximated from the Gauss–Newton Hessian as

(29) $$\Sigma_{\xi }=\lambda {\left(J_{\xi }^{T}\Sigma_{\epsilon }^{-1}J_{\xi }\right)}^{-1},\;\lambda =\frac{r_{B}^{T}\Sigma_{\epsilon }^{-1}r_{B}}{3L},$$

where *L* is the number of magnetometers in the array. The covariance of the accumulated magnetic relative-pose measurement between two image frames is then used as $\Sigma_{k}^{k+n}$ in the backend factor, and its inverse acts as the information matrix of the magnetic relative pose factor.

Here, $x_{k}$ denotes the state of the carrier and $e_{k}^{k+n}$ denotes the residual, which is the difference between the relative pose measurement $z_{k}^{k+n}$ and the value of the measurement equation $h(x_{k},x_{k+n})$. In the relative pose factor, the measurement equation can be expressed as:

(30) $$h\left(x_{k},x_{k+n}\right)={T_{k}^{w}}^{-1}T_{k+n}^{w}.$$

Here, $T_{k}^{w}$ denotes the pose transformation from the body frame to the world frame.

Ordinarily, the residual is conventionally defined as z−h(x); however, for the relative-pose factor, the residual corresponds to the difference between poses. Since the pose matrices belong to the special Euclidean group SE(3), they do not exhibit closure under addition. Moreover, directly defining an error on SE(3) lacks both mathematical validity and the capacity to quantify the actual error magnitude. Accordingly, it is necessary to map the pose error from SE(3) to its tangent space se(3) by means of the logarithmic map:

(31) $$e_{k}^{k+n}=\mathrm{Log}{\left({\left(z_{k}^{k+n}\right)}^{-1}\left({T_{k}^{w}}^{-1}T_{k+n}^{w}\right)\right)}^{\vee }.$$

### 5.3. Reprojection Factor

Similar to the relative pose factor, the core of constructing the reprojection factor also lies in error evaluation. Specifically, the reprojection factor primarily evaluates the difference between two positions on the image plane: one is the projected position of the 3D position state of feature points (after transformation via the carrier’s pose state), and the other is the actual position of the feature points on the image plane. The measurement equation can be expressed as follows:

(32) $$h\left(x_{k},l_{j}\right)=\pi \left(K\left[I_{3}\;0\right]{T_{k}^{w}}^{-1}{\bar{l}}_{j}\right).$$

Here, $l_{j}$ denotes the state of the *j*th 3D feature point, ${\bar{l}}_{j}$ is its homogeneous form, and π(·) denotes the homogeneous projection from the camera coordinate frame to the image plane.

From (32), it can be inferred that the reprojection factor simultaneously constrains both the feature point states and the carrier pose states. These two types of states will be optimized concurrently within the factor graph.

## 6. Experiments

In this section, we evaluate the proposed odometry system under a synthesized magnetic field setting. The experiments are organized at two levels. First, frontend-level simulations are conducted to analyze how the magnetometer array configuration and calibration residuals influence magnetic relative-pose estimation and the covariance information used by the backend magnetic factor. Then, trajectory-level evaluations are performed on selected EuRoC sequences with simulated magnetic measurements, including localization accuracy comparison with two reference odometry baselines and computational load evaluation of the proposed method. This evaluation is intended to assess the feasibility and potential benefit of introducing magnetic constraints into visual odometry.

### 6.1. Magnetic Frontend Analysis

In a simulated magnetic field, a set of frontend-level analyses is conducted to examine how magnetometer array configuration and calibration residuals affect magnetic relative-pose estimation before the full visual-magnetic backend optimization. The analysis focuses on three factors: the spatial configuration of the array, the number of magnetometers, and the sensitivity of the magnetic frontend to calibration errors. The resulting frontend accuracy and uncertainty provide an intermediate link between these frontend configuration factors and the expected behavior of the complete MVO system.

#### 6.1.1. Effect of Sensor Spatial Configuration

**Figure 4:**
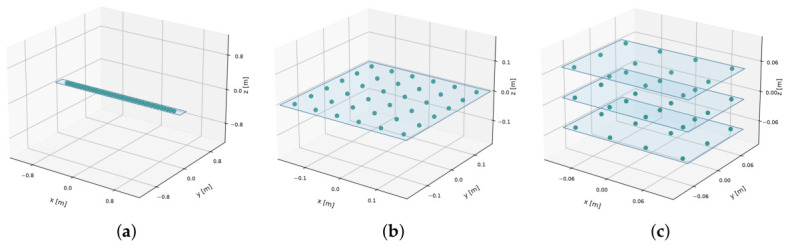
Sensor spatial configurations used in the magnetic frontend analysis. (**a**) Line array (36×1×1). (**b**) Square planar array (6×6×1). (**c**) 3D array (4×3×3).

We first evaluate how the spatial configuration of the magnetometer array affects the accuracy of the magnetic frontend. This frontend-level simulation is intended to isolate the influence of array geometry from the visual frontend and backend optimization. The reference array scale and the 60 mm inter-sensor pitch are chosen according to the magnetometer array configuration used in MAINS [19]. To make the comparison controlled while allowing regular line, planar, and volumetric layouts, the three layouts use the same number of magnetometers (L=36) and the same inter-sensor pitch. The tested layouts include a one-dimensional line array (36×1×1), a square planar array (6×6×1), and a three-dimensional array (4×3×3), as shown in Figure 4. For each layout, three short straight trajectories are generated from the same initial pose, with a total translation of 150 mm along the *x*, *y*, or *z* direction. The single-axis motions separately excite local magnetic field variations along the three principal directions, making it possible to observe how different array configurations constrain motion in each direction. The short trajectory setting also reduces the influence of trajectory length, accumulated drift, and path curvature on the comparison of array geometry. The magnetic frontend is evaluated using the initial-frame-aligned position RMSE and orientation RMSE over each trajectory.

**Figure 5:**
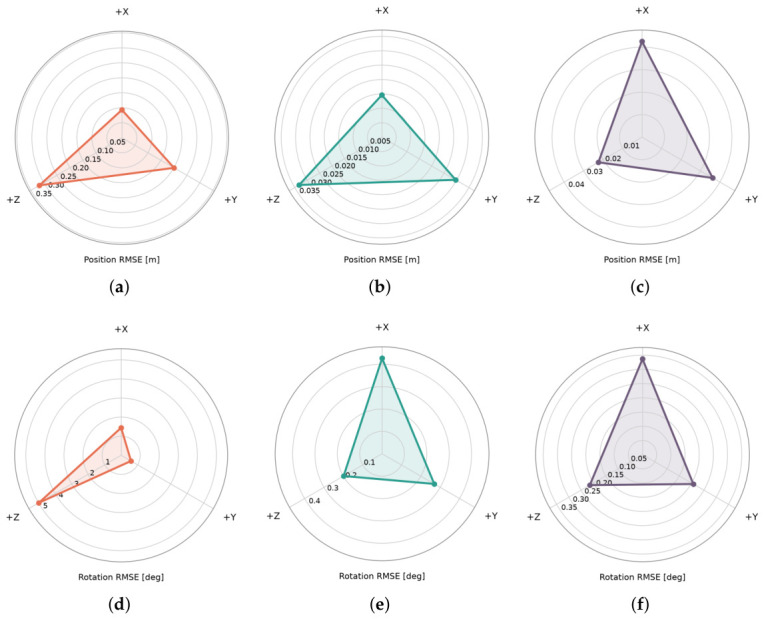
Initial-frame-aligned trajectory accuracy under different spatial configurations. The first row reports position RMSE and the second row reports orientation RMSE. (**a**,**d**) Line array. (**b**,**e**) Square planar array. (**c**,**f**) 3D array.

Figure 5 shows that the line array has the weakest overall performance. Its average position RMSE is 0.2038 m and its average orientation RMSE is 2.3431 deg, which are much larger than those of the planar and 3D layouts. This degradation is especially evident when the motion direction is poorly constrained by the one-dimensional sensor distribution, because the array cannot sufficiently sample magnetic field variations in multiple spatial directions.

The square planar array provides the lowest average position RMSE, 0.0258 m, and maintains a low orientation RMSE of 0.2982 deg. The 3D array achieves the lowest average orientation RMSE, 0.2528 deg, but its average position RMSE, 0.0337 m, is slightly higher than that of the planar array. Under a fixed total number of sensors, distributing sensors in three dimensions can improve orientation observability, but it also reduces the sampling density on each local plane. Therefore, in the tested short-range trajectory-estimation setting, the planar array provides a better balance between position accuracy, orientation accuracy, installation simplicity, and calibration complexity. This result supports the use of a planar magnetometer array in the current MVO prototype, while 3D configurations may still be useful when strong three-dimensional rotational motion or volumetric installation space is available.

#### 6.1.2. Effect of Sensor Number

**Figure 6:**
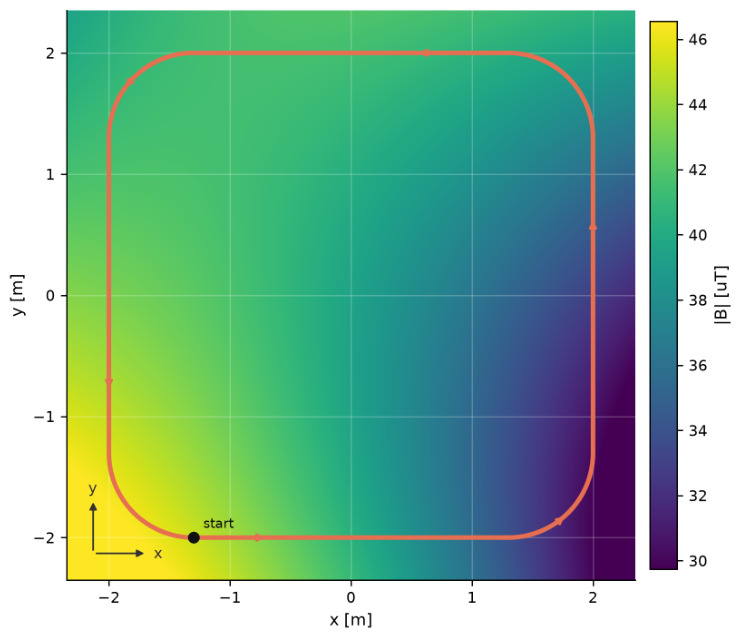
Rounded-square trajectory and simulated magnetic field environment used in the sensor number analysis.

**Figure 7:**
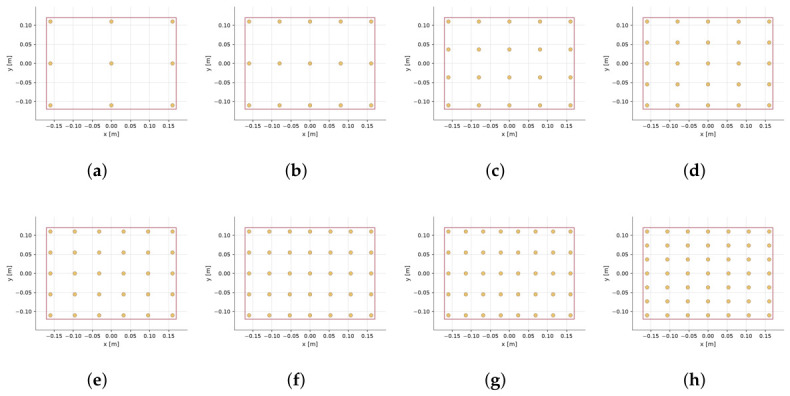
Planar magnetometer layouts used in the sensor-number analysis. The red rectangle indicates the sensor boundary. (**a**) L=9. (**b**) L=15. (**c**) L=20. (**d**) L=25. (**e**) L=30. (**f**) L=35. (**g**) L=40. (**h**) L=49.

After the spatial configuration analysis, the planar array is adopted as the reference layout for the subsequent frontend simulations. We then evaluate how the number of magnetometers affects the magnetic frontend under a fixed planar array size. Unlike the preceding short single-axis tests, this experiment uses a rounded-square trajectory in the simulated magnetic field environment, as shown in Figure 6, so that the magnetic frontend is tested over multiple motion directions and spatial locations. The array footprint is kept within approximately 0.32m×0.22m, and different numbers of magnetometers are distributed as uniformly as possible on the same plane. The tested sensor numbers are L=9, 15, 20, 25, 30, 35, 40, and 49, as shown in Figure 7. For each sensor number, 30 independent Monte Carlo trials are performed by injecting independent Gaussian magnetic measurement noise with a standard deviation of 15nT, and the magnetic frontend is evaluated using the initial-frame-aligned position RMSE and orientation RMSE. This setting is used to examine the statistical influence of sensor density under random measurement noise.

**Figure 8:**
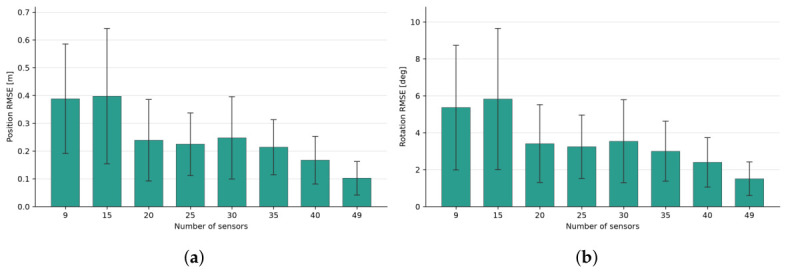
Magnetic frontend accuracy under different magnetometer numbers with 30 Monte Carlo trials. (**a**) Initial-frame-aligned position RMSE. (**b**) Initial-frame-aligned orientation RMSE.

As shown in Figure 8, increasing the number of magnetometers generally improves both position and orientation accuracy, but the improvement is not strictly monotonic in the low-to-middle range. With 9 and 15 magnetometers, the frontend produces relatively large errors and fluctuations, with position RMSEs of 0.3880±0.1969 m and 0.3975±0.2442 m, and orientation RMSEs of 5.359±3.371 deg and 5.821±3.830 deg, respectively. This indicates that a sparse planar array is insufficient to stably sample the local magnetic field variation under measurement noise.

When the number of magnetometers increases to 20–35, the frontend enters a more usable range, although the results still fluctuate due to the random noise realization and the discrete layout. The position RMSEs in this range are between 0.2137 m and 0.2473 m, and the orientation RMSEs are between 2.995 deg and 3.537 deg. Lower errors are obtained with 40 and 49 magnetometers. In particular, the 49-magnetometer array achieves a position RMSE of 0.1019±0.0601 m and an orientation RMSE of 1.505±0.911 deg, corresponding to reductions of approximately 73.7% and 71.9%, respectively, compared with the 9-magnetometer case. These results suggest that increasing the sensor density within a fixed planar array improves the robustness of magnetic relative-pose estimation. For the subsequent trajectory-level MVO evaluation, we adopt the 30-magnetometer planar configuration as the reference array setting. This choice follows the array scale used in previous magnetic-aided navigation work and keeps the trajectory-level evaluation based on a representative magnetometer-array size. This configuration provides sufficient frontend accuracy for the feasibility evaluation in this paper, although it is not the lowest-error configuration in the above simulation. The sensor-number analysis further indicates that increasing the sensor density can improve the magnetic frontend statistically, while the non-monotonic behavior in the middle range shows that the array configuration should be interpreted together with sensor number, discrete spatial layout, and magnetic field variation.

#### 6.1.3. Effect of Calibration Error

Based on the preceding two analyses, the calibration error simulation uses the planar array with 30 magnetometers as the reference frontend configuration. This experiment evaluates how representative calibration residuals affect the magnetic frontend and the covariance passed to the backend magnetic relative-pose factor. Four types of calibration residuals are considered: hard-iron bias, scale/soft-iron residual, sensor-position misalignment, and sensor-orientation misalignment. Each residual type is injected separately with one baseline level and three increasing levels. Let $B(r_{i,k})$ be the simulated magnetic field at the true position of the *i*-th magnetometer at frame *k*, and let $n_{i,k}$ be Gaussian measurement noise. The four corrupted measurements are defined as

(33a) $$\begin{array}{cccc} {\tilde{b}}_{i,k}^{h} & =B\left(r_{i,k}\right)+\Delta h_{i}+n_{i,k},\; & \Delta h_{i} & ∼N(0,\sigma_{h}^{2}I_{3}), \end{array}$$

(33b) $$\begin{array}{cccc} {\tilde{b}}_{i,k}^{s} & =\left(I+\Delta S_{i}\right)B\left(r_{i,k}\right)+n_{i,k},\; & \Delta S_{i} & =\mathrm{diag}\left(s_{ix},s_{iy},s_{iz}\right),\;s_{ij}∼N\left(0,\sigma_{s}^{2}\right), \end{array}$$

(33c) $$\begin{array}{cccc} {\tilde{b}}_{i,k}^{p} & =B\left(r_{i,k}+\Delta s_{i}\right)+n_{i,k},\; & \Delta s_{i} & ∼N(0,\sigma_{p}^{2}I_{3}), \end{array}$$

(33d) $$\begin{array}{cccc} {\tilde{b}}_{i,k}^{\theta } & =R\left(\Delta \theta_{i}\right)B\left(r_{i,k}\right)+n_{i,k},\; & \Delta \theta_{i} & ∼N(0,\sigma_{\theta }^{2}I_{3}). \end{array}$$

Here, the superscripts *h*, *s*, *p*, and θ denote hard-iron bias, scale/soft-iron residual, sensor-position misalignment, and sensor-orientation misalignment, respectively. The baseline/low/medium/high levels are defined by the following standard deviations:

(34) $$\begin{array}{cccc} \sigma_{h} & \in \{0,0.25,0.50,0.75\}\;\mu T, & \sigma_{s} & \in \{0,0.5,2,5\}\%,\\ \sigma_{p} & \in \{0,0.5,1.0,1.5\}\;\mathrm{mm}, & \sigma_{\theta } & \in \{0,0.5,1.0,1.5\}\;\deg. \end{array}$$

The rounded-square trajectory and simulated magnetic field environment shown in Figure 6 are used again, and 30 Monte Carlo trials are performed for each error type and level with 15nT Gaussian magnetic measurement noise. The frontend trajectory accuracy is evaluated using the initial-frame-aligned position RMSE and orientation RMSE.

**Figure 9:**
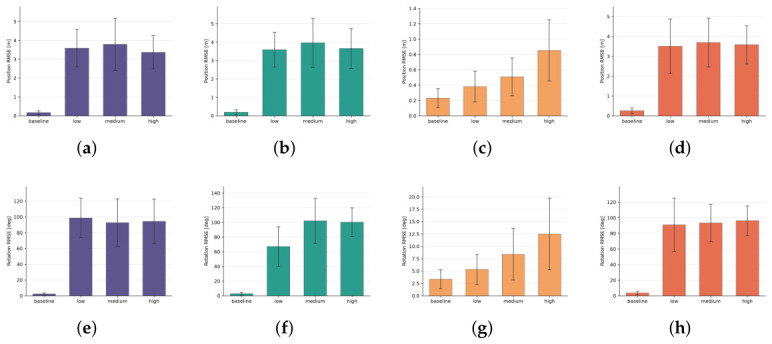
Frontend trajectory accuracy under calibration-error injection with 30 Monte Carlo trials. The first row reports initial-frame-aligned position RMSE and the second row reports initial-frame-aligned orientation RMSE. (**a**,**e**) Hard-iron bias. (**b**,**f**) Scale/soft-iron residual. (**c**,**g**) Sensor-position misalignment. (**d**,**h**) Sensor-orientation misalignment.

Figure 9 shows that calibration residuals that directly distort the measured three-axis magnetic vector can cause clear frontend-level degradation. Hard-iron bias increases the position RMSE from the baseline level of about 0.17 m to approximately 3.37–3.79 m, and increases the orientation RMSE to approximately 92–99 deg. Scale/soft-iron residuals lead to a similar degradation, with position RMSEs of approximately 3.59–3.96 m and orientation RMSEs of approximately 67–102 deg. Sensor-orientation misalignment also strongly affects the frontend estimate: the low/medium/high levels produce position RMSEs of approximately 3.50–3.69 m and orientation RMSEs of approximately 91–96 deg. In contrast, sensor-position misalignment produces a weaker but still visible degradation, with position RMSE increasing from about 0.38 m at 0.5 mm to about 0.85 m at 1.5 mm, and orientation RMSE increasing from about 5.31 deg to about 12.48 deg.

To connect the frontend degradation to the backend factor weight, the full 6-D pose covariance is further analyzed. Consistent with the covariance definition in the backend section, the frontend covariance is approximated from the Gauss–Newton linearization as $\lambda {\left(J^{⊤}\Sigma^{-1}J\right)}^{-1}$, with the residual-based scale factor λ and the perturbation order ${\left[\delta \phi^{⊤},\delta t^{⊤}\right]}^{⊤}$. In the backend, this covariance is used to construct the information matrix of the magnetic relative-pose factor, so both diagonal and off-diagonal covariance terms affect the effective weighting of the residual.

**Figure 10:**
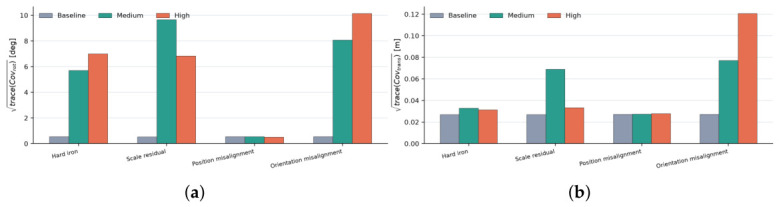
Covariance trace statistics under calibration-error injection. (**a**) Square root of the rotational covariance trace. (**b**) Square root of the translational covariance trace.

As shown in Figure 10, different calibration residuals are reflected differently in the frontend covariance. Hard-iron bias mainly increases the rotational covariance, while the translational covariance changes only mildly. Scale/soft-iron residuals and sensor-orientation misalignment increase both rotational and translational uncertainty, so the backend can reduce the overall weight of the affected magnetic factor when the full covariance is used. Sensor-position misalignment is more difficult to detect from the current covariance model: although it degrades the trajectory accuracy, the covariance trace changes weakly because the uncertainty of the sensor positions is not explicitly propagated into the magnetic measurement covariance.

**Figure 11:**
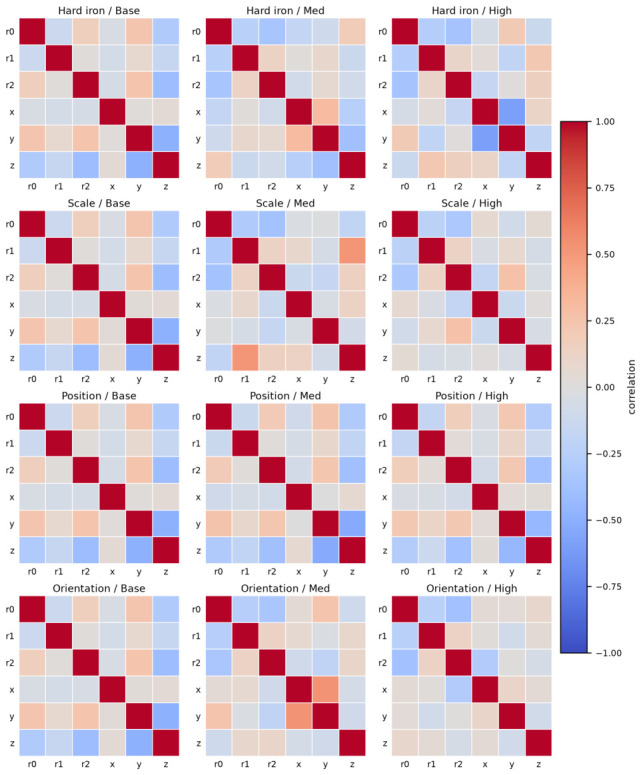
Mean correlation matrices of the full 6-D frontend covariance under calibration error injection.

Figure 11 further shows that the frontend covariance is not strictly diagonal. The off-diagonal terms describe correlations among rotational axes, translational axes, and rotation–translation components. Therefore, using the full 6×6 covariance in the backend is more appropriate than assigning independent scalar weights to each degree of freedom. However, the position-misalignment case also shows a limitation of the current covariance formulation: systematic geometric calibration residuals may reduce the true trajectory accuracy without being fully represented in the local covariance. This indicates that magnetic array calibration quality directly affects not only frontend trajectory accuracy but also the reliability of the covariance used by the backend magnetic factor.

The calibration residuals analyzed above directly perturb the magnetic array observation model. The camera–magnetometer extrinsic calibration error belongs to a different level of the system. It mainly affects the transformation of the magnetic relative-pose estimate into the camera/visual frame, and its influence is then propagated to magnetic-aided feature prediction and backend visual–magnetic fusion. Therefore, real hardware extrinsic calibration and its coupled influence on the complete MVO system will be evaluated in future real-array experiments.

### 6.2. Trajectory-Level Evaluation

#### 6.2.1. Evaluation Setup

As an initial simulation-based feasibility evaluation, the experimental evaluations in this paper are conducted on a public UAV (unmanned aerial vehicle) visual dataset with a simulated magnetic field superimposed. The public dataset selected herein is the EuRoC dataset, which contains three distinct scenarios and 11 data sequences. Each data sequence includes onboard sensor data and the ground-truth trajectory data of the UAV.

**Figure 12:**
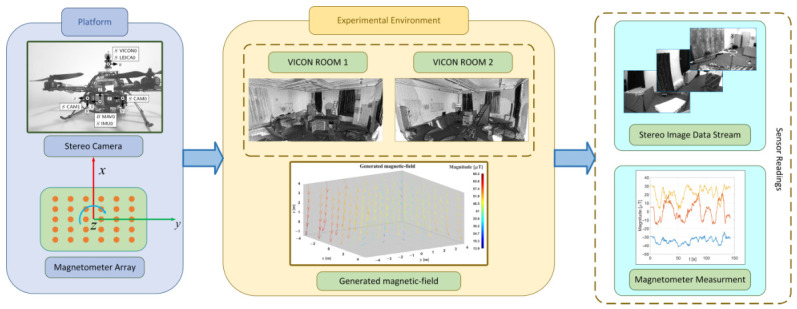
Experimental platform, environment, and sensor readings.

As shown in Figure 12, the carrier platform employed in the experiment is a quadrotor UAV, which is equipped with a stereo camera. Its parameters are detailed in [23]. The magnetic field acquisition sensor is a sensor array composed of 30 3-axis magnetometers, with parameters conforming to the RM3100 standard. This magnetometer features a measurement range of ±800μT, a sampling rate of 200 Hz, and a noise level of 15nT.

As mentioned earlier, the experiments in this paper are conducted on a public UAV visual dataset with a simulated magnetic field superimposed. As shown in the experimental environment in Figure 12, we selected two scenarios with definite spatial dimensions from the EuRoC dataset: Vicon Room 1 and Vicon Room 2. As noted in [23], these two scenarios have a spatial dimension of 8 m × 8.4 m × 4 m; based on this spatial dimension, the indoor magnetic field was simulated within the experimental space.

When creating the simulated magnetic field, we simulate the inhomogeneous magnetic field caused by ferromagnetic interference in indoor environments by placing magnetic dipoles around the experimental space, such that the magnetic fields generated by these multiple dipoles superimpose within the experimental space. The magnetic field strength H of a magnetic dipole with a magnetic moment m at position r is given by:

(35) $$H=\frac{1}{4\pi }\left(\frac{3(r\cdot m)r}{{\left|r\right|}^{5}}-\frac{m}{{\left|r\right|}^{3}}\right).$$

**Figure 13:**
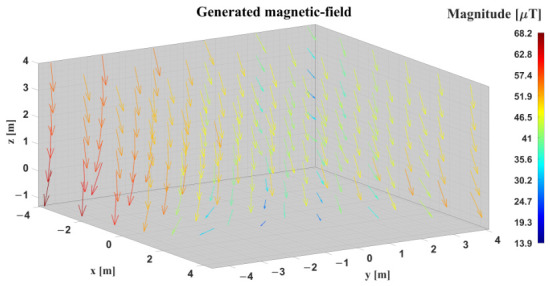
Vector diagram of magnetic field distribution in the experimental space.

Furthermore, a uniform magnetic field was also superimposed on the experimental space to simulate the geomagnetic field. Finally, this resulted in the formation of an indoor spatial magnetic field within the experimental space, as shown in Figure 13. In the figure, the distribution of the intensity and direction of the simulated indoor magnetic field is visualized using colored arrows. The magnetic field intensity ranges from 12μT to 70μT, which is consistent with the intensity range of most indoor scenarios.

**Figure 14:**
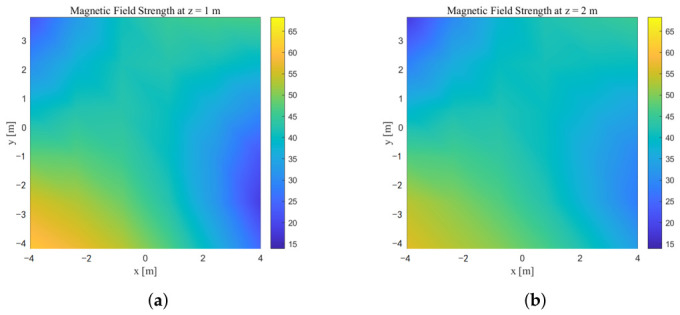

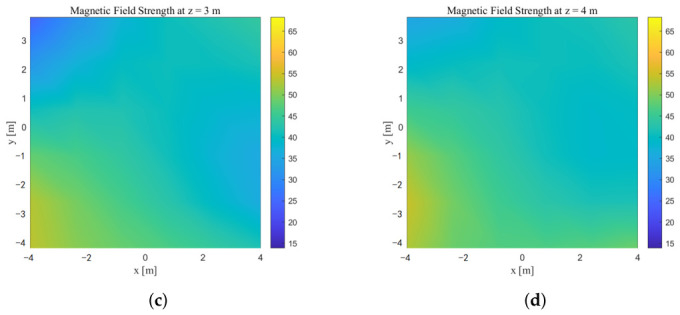
Magnetic field distribution on planes at different heights in the experimental space: (**a**) z=1m, (**b**) z=2m, (**c**) z=3m, (**d**) z=4m.

To more clearly illustrate the distribution of the simulated magnetic field intensity, we plotted the magnetic field intensity distributions of four planes at different heights within the experimental space in Figure 14. It can be observed that the magnetic field gradient is relatively large in regions near the boundaries of the experimental area in the figure, which is consistent with the scenario in actual buildings where relatively large magnetic field gradients are induced by the presence of ferromagnetic materials (e.g., steel bars) in wall structures.

Once the simulated magnetic field is constructed, we obtain a complete experimental environment, as shown in Figure 12. The sensor data comprise stereo image stream data and magnetic field vector data, and the stereo image stream data—collected by the carrier during its flight within the experimental environment—are already included in the EuRoC dataset; meanwhile, the measurement values of the magnetometer array sensor are obtained by moving the magnetometer array in accordance with the ground-truth trajectory provided in the dataset during the simulation.

#### 6.2.2. Localization Accuracy

We compare the proposed MVO with two reference odometry baselines: MSCKF-Stereo and VINS-Fusion. MSCKF-Stereo is a filter-based stereo visual-inertial odometry (VIO) method derived from the multi-state constraint Kalman filter (MSCKF) framework, and it is designed for robust and efficient state estimation using stereo cameras and inertial measurements [11,15]. VINS-Fusion is an open-source optimization-based multi-sensor state estimation framework built on the VINS family; its local visual-inertial estimator is based on nonlinear optimization with visual reprojection and IMU preintegration constraints [8,24]. In this work, loop closure is disabled in VINS-Fusion so that the comparison focuses on odometry rather than global relocalization or map reuse. These two methods are selected as representative filtering-based and optimization-based VIO reference baselines under the same EuRoC visual data and trajectory, while the proposed MVO uses the same visual data together with synthesized magnetic measurements.

Localization accuracy evaluation of the three aforementioned methods was conducted on the EuRoC dataset with superimposed simulated magnetic fields. Specifically, MSCKF-Stereo and VINS-Fusion utilize the image and IMU data from the dataset, while MVO leverages the dataset’s image data and simulation-generated magnetic field measurement data. The data employed comprises two scenarios—Vicon Room 1 and Vicon Room 2—with a total of six data sequences. Each scenario contains data sequences corresponding to three distinct operating conditions, where differences in operating conditions refer to variations in the UAV’s movement speed and the quality of the captured images. The specific operating conditions are detailed in Table 1. For the accuracy evaluation, the open-source evaluation tool evo was employed. Additionally, the root mean square error (RMSE) of the absolute trajectory error (ATE) was selected as the key evaluation metric.

The localization accuracy results are presented in Table 2, where the best values are highlighted in bold. MSCKF-Stereo and VINS-Fusion exhibit mixed performance across the evaluated sequences. MSCKF-Stereo fails on V2-03, where significant illumination variations can degrade LKT optical-flow tracking and leave insufficient features to constrain the filter. VINS-Fusion also uses LKT tracking, but its optimization-based fusion can provide stronger constraints against transient visual degradation. Under the synthesized magnetic field setting, MVO achieves the lowest RMSE ATE in five out of the six sequences, suggesting that the added magnetic relative-pose constraints can improve the proposed MVO under the tested setting. This comparison should be interpreted as a reference evaluation with MSCKF-Stereo and VINS-Fusion under the same visual data and trajectory, rather than as a full hardware-level comparison between real magnetometer arrays and real IMU systems.

**Figure 15:**
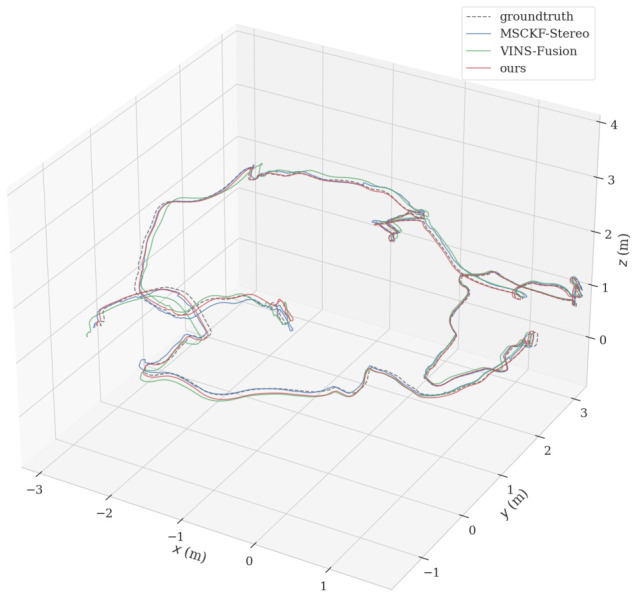
Trajectories produced by MSCKF-Stereo, VINS-Fusion, and MVO on the V2-01 sequence of the EuRoC dataset.

To visualize the trajectory-level behavior, Figure 15 presents the estimated trajectories of the three methods on the V2-01 sequence, where all three methods complete the sequence. The overall trajectory trends are similar, while MVO remains closest to the ground truth in this case.

**Figure 16:**
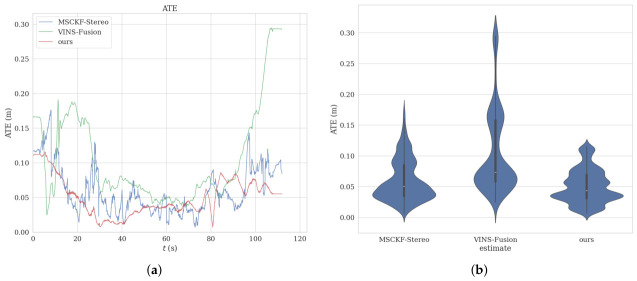
Absolute trajectory error comparison between MVO and the baseline methods. (**a**) Temporal evolution of ATE. (**b**) Violin plots.

Figure 16a presents the temporal distribution of the absolute trajectory error (ATE) of the three systems on V2-01, and Figure 16b shows the corresponding violin plots. MVO has fewer large-error samples, a lower median error, and an error distribution more concentrated in the low-error region.

#### 6.2.3. Computational Load

In addition to localization accuracy, the computational load of the proposed method was evaluated because real-time operation is essential for indoor robotic applications. Before presenting the measured results, we first analyze the main load components of MVO and the two baseline methods. The computational load of MVO can be decomposed as

(36) $$C_{\mathrm{MVO}}=C_{\mathrm{vis}}+C_{\mathrm{mag}}+C_{\mathrm{opt}}\left(6N\right),$$

where $C_{\mathrm{vis}}$ denotes the visual frontend load, $C_{\mathrm{mag}}$ denotes the magnetic relative-pose estimation load, and $C_{\mathrm{opt}}\left(6N\right)$ denotes the backend optimization load with *N* pose states. The magnetic frontend operates on a fixed-size magnetometer array and a local magnetic field model. With the number of magnetometers and the model dimension fixed in the system, this module introduces a bounded per-frame computation rather than a load that grows with the trajectory length.

For a typical visual-inertial optimization framework, the computational load can be written as

(37) $$C_{\mathrm{VIO}}=C_{\mathrm{vis}}+C_{\mathrm{imu}}+C_{\mathrm{opt}}\left(15N\right),$$

where $C_{\mathrm{imu}}$ includes IMU preintegration and inertial covariance propagation. The backend state of a VIO system usually contains pose, velocity, gyroscope bias, and accelerometer bias for each keyframe, resulting in approximately 15 state variables per keyframe. In contrast, MVO introduces the magnetic measurement as a relative pose factor between consecutive poses and does not augment each keyframe with velocity or inertial bias states. Therefore, compared with an optimization-based VIO method such as VINS-Fusion, MVO has a lighter backend state representation. However, compared with the filtering-based MSCKF-Stereo, MVO still includes an optimization backend and an additional magnetic frontend, so its computational load is expected to be higher than that of the recursive filter-based baseline.

**Figure 17:**
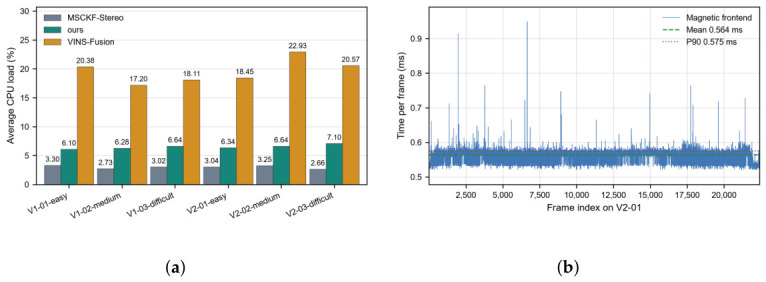
Computational load evaluation. (**a**) Average CPU load of MSCKF-Stereo, VINS-Fusion, and MVO on the Vicon Room sequences under rosbag replay. (**b**) Per-frame runtime of the single-thread magnetic frontend on the V2-01 sequence.

All computational-load experiments were conducted on a mini computer equipped with an AMD Ryzen 9 7940HS processor with Radeon 780M integrated graphics (8 cores and 16 threads) and 32 GB RAM, running Ubuntu 20.04 LTS 64-bit. No GPU acceleration was used in the compared methods. The three methods were further tested using the same Vicon Room sequences under ROS-based rosbag replay, and the average CPU load was recorded for each sequence. For MVO, the per-frame runtime of the single-thread magnetic frontend was additionally recorded on the V2-01 sequence. Figure 17 presents the computational load evaluation results. Across the six evaluated sequences, MSCKF-Stereo had the lowest average CPU load of 3.00%, which is consistent with its filtering-based formulation and lightweight recursive update. VINS-Fusion required the highest average CPU load of 19.61%, mainly because its optimization-based visual-inertial formulation includes IMU preintegration and augments each keyframe state with velocity and inertial bias variables. The proposed MVO achieved an average CPU load of 6.52%, which is 66.8% lower than that of VINS-Fusion, but about 2.17 times higher than that of MSCKF-Stereo.

The per-frame runtime curve in Figure 17b further supports the above analysis. On the V2-01 sequence, the single-thread magnetic frontend required 0.564 ms per frame on average, with a 90th percentile of 0.575 ms and a maximum value below 1 ms. This result indicates that the magnetic frontend introduces a fixed and lightweight additional load in the tested setting.

## 7. Conclusions

In this study, a magneto-visual odometry (MVO) system is presented as an initial simulation-based feasibility study for integrating indoor magnetic field anomalies with visual information in GNSS-denied environments. By utilizing a 30-magnetometer planar array model, the system fits a polynomial-based local magnetic field model to estimate relative poses between consecutive measurements. These magnetic-derived results provide motion priors that assist the visual frontend in feature matching, thereby improving tracking continuity during visual degradation. A factor graph optimization (FGO) framework is established to fuse the magnetic relative-pose factors and visual reprojection factors, while the iSAM2 algorithm is employed to ensure efficient incremental optimization.

The experiments evaluate the method at both the frontend and trajectory levels. The magnetic frontend analysis shows that sensor spatial configuration, sensor number, and calibration residuals all affect frontend position/orientation accuracy and the covariance used by the backend magnetic factor. These results support the use of the 30-magnetometer planar array as the reference configuration in the trajectory-level evaluation and highlight the importance of magnetometer-array calibration. On the EuRoC MAV dataset augmented with high-fidelity synthesized magnetic field data, the trajectory-level results indicate the potential localization benefit of adding magnetic constraints compared with the selected reference baselines, while the computational load evaluation shows that the proposed method remains lighter than VINS-Fusion but heavier than the filtering-based MSCKF-Stereo baseline.

These results support the feasibility of the proposed magneto-visual formulation, but they do not replace validation with real magnetometer array measurements. Future research will focus on real-array deployment, including magnetometer-array calibration, camera–magnetometer extrinsic calibration, real magnetic-visual data acquisition, and evaluation on a dedicated hardware platform in diverse indoor environments.

## Figures and Tables

**Table 1:** Dataset characteristics.

Name	Length/	Avg. Vel./	Note
	**Duration**	**Angular Vel.**	
V1-01	58.6 m	0.41 m/s	slow motion,
	144 s	0.28 rad/s	bright scene
V1-02	75.9 m	0.91 m/s	fast motion,
	83.5 s	0.56 rad/s	bright scene
V1-03	79.0 m	0.75 m/s	fast motion,
	105 s	0.62 rad/s	motion blur
V2-01	36.5 m	0.33 m/s	slow motion,
	112 s	0.28 rad/s	bright scene
V2-02	83.2 m	0.72 m/s	fast motion,
	115 s	0.59 rad/s	bright scene
V2-03	86.1 m	0.75 m/s	fast motion,
	115 s	0.66 rad/s	motion blur

**Table 2:** Performance comparison on all evaluated sequences (RMSE ATE in meters).

Sequence	V1-01	V1-02	V1-03	V2-01	V2-02	V2-03
MSCKF-Stereo	0.157	0.156	0.221	0.069	0.139	fail *
VINS-Fusion	0.192	**0.125**	0.197	0.126	0.149	0.344
**Ours (MVO)**	**0.095**	0.137	**0.163**	**0.050**	**0.128**	**0.254**

Bold values indicate the lowest RMSE ATE for each sequence. * The estimator lost tracking or diverged before completing the sequence, so a valid full-trajectory RMSE ATE could not be computed.

## Data Availability

The EuRoC dataset used in this study is available at https://www.research-collection.ethz.ch/entities/researchdata/bcaf173e-5dac-484b-bc37-faf97a594f1f. The simulated magnetic field data used in this study are available from the corresponding author upon reasonable request.
